# Estimating the total prevalence of PTSD among the UK police force: Formal comment on Stevelink et al. (2020)

**DOI:** 10.1371/journal.pone.0268621

**Published:** 2022-05-20

**Authors:** Chris R. Brewin, Jessica K. Miller, Brendan Burchell

**Affiliations:** 1 Clinical, Educational & Health Psychology, University College London, London, United Kingdom; 2 Department of Sociology, University of Cambridge, Cambridge, United Kingdom; University of the Witwatersrand, SOUTH AFRICA

## Abstract

Two recent surveys have reported widely differing prevalence rates for posttraumatic stress disorder (PTSD) within the U.K. police force. Stevelink et al. (2020) reported a rate of 3.9% whereas a survey conducted for the charity Police Care UK reported a rate of 20.6%. In this comment we discuss how definitions and methodological factors can impact prevalence rates. We consider a number of possible reasons for the discrepancy between the surveys, and conclude that it is most likely a method artefact. Stevelink et al.’s survey reported the prevalence of recent-onset DSM-IV PTSD only, whereas the Police Care UK survey reported the total ICD-11 PTSD and Complex PTSD prevalence, regardless of when in the person’s career the traumatic events occurred. Analysing the Police Care UK data using Stevelink et al.’s procedures produced practically identical prevalence rates, suggesting that the discrepancy was apparent rather than real.

*PLOS ONE* recently published a large survey by Stevelink and colleagues of psychological ill-health involving 40,299 U.K. police officers and staff [1]. The authors reported that they had found a probable DSM-IV posttraumatic stress disorder (PTSD) prevalence of 3.9% which they suggested was likely to be more accurate than a prevalence of ICD-11 PTSD plus Complex PTSD of 20.6% found in another recent study of 10,401 U.K. police officers conducted on behalf of the charity Police Care UK [2]. The existence of this large discrepancy is extremely important for public policy and could have major implications for the future resourcing of occupational health services for the police, both in the U.K. and elsewhere. In this commentary we discuss how different survey methods, as well as different types of prevalence, may impact reported rates of conditions such as PTSD. We look further into the survey conducted by Stevelink et al. and conclude that the apparent discrepancy is an artefact due to different ways of measuring prevalence.

Differences in prevalence may sometimes be attributable to the use of different diagnostic systems. Successive versions of the Diagnostic and Statistical Manual published by the American Psychiatric Association have not identified a separate diagnosis of Complex PTSD. The latest edition of the International Classification of Diseases (ICD-11) published by the World Health Organization does however distinguish between PTSD and Complex PTSD [3]. A considerable number of studies have now compared prevalence rates using the two systems showing that rates of PTSD using DSM-based diagnoses are generally slightly higher than those combining ICD-11 PTSD and Complex PTSD [4]. Differences in the measures used do not therefore provide a plausible explanation for the higher rates in the Police Care UK survey, subsequently published in Psychological Medicine [5].

One explanation for discrepant rates is that some studies are framed as trauma or mental health surveys whereas others are not. It is reasonable to suppose that officers with mental health problems or trauma issues might be more likely to opt in to surveys that are framed in this way, thereby inflating prevalence rates. Stevelink et al. suggested that the Police Care UK survey [2], subsequently published in Psychological Medicine [5], was framed as a trauma or mental health survey whereas theirs was not. Characterising the framing of a survey is, however, not straightforward.

One issue is that the topic of trauma can be presented in ways that either emphasise vulnerability and mental health issues, or in more positive ways that emphasise training and resilience. It can also be a main or a subsidiary focus of the survey. The Police Care UK survey, for example, covered numerous aspects of officers’ views about their working environment. Although trauma was included in this, its title "Policing: The Job & The Life" was chosen so as not to emphasise a mental health focus.

Today, large-scale surveys are often advertised online via social media campaigns. The involvement of social media, in which messages are delivered in different ways and passed on via different pathways such as re-tweeting, means that the original framing by the survey authors may become diluted or altered in the process. As a consequence, surveys should monitor social media messaging to detect the presence of any “drift” from the way it was originally presented. For example, the campaign to advertise the "Policing: The Job & The Life" survey was conducted via Facebook and Twitter. Of the tweets, some were trauma-specific, some made no reference to trauma at all, and some referred to trauma in a neutral or positive context (such as trauma management and trauma resilience) or alongside other subjects and topics, ranging from sleep patterns to pride in the job. These different tweets were re-tweeted at different rates. Five posts were produced on Facebook, variously making no reference to trauma at all, focusing on managing resilience, or in relation to management and presenteeism. These posts were in turn shared at different rates.

These observations indicate that there may be considerable variability in how potential respondents initially come across and understand the nature of an online survey, and how messages may change over time. Framing also offers the opportunity for selection bias in other ways. For example, recruitment to the second phase of the Stevelink et al. (2020) study was “via publicity of a full, free health screen as well as participation in a major research project on the health of police employees”. As the authors acknowledge, it seems quite possible that this may also have resulted in some selective recruitment of employees with concerns about their physical or mental health. A final point is that ethical considerations require respondents to have a more detailed awareness of survey contents before they agree to participate. So self-selection may occur, not just when the person initially hears about the survey, but when this more detailed information is presented.

There are other aspects of survey methodology that may be important in understanding why studies achieve different prevalence rates. For example, PTSD rates tend to be lower in non-anonymous surveys, an effect that is generally attributed to stigma suppressing true reporting [6].

There is another major factor to consider in explaining discrepancies in PTSD rates, and this is the way in which exposure to traumatic events is assessed. PTSD is assessed differently to other disorders in epidemiological surveys because there has to be a qualifying traumatic event or events and symptoms are established in relation to this. Typically respondents are asked about the occurrence of potentially traumatic events over extended periods such as the lifetime [7,8] or military deployment to certain theatres [9]. Such measurement is subject to some variability over successive occasions and may be influenced by current mood, with positive mood associated with reduced reporting of events and negative mood with greater reporting [10,11].

Nevertheless, the choice of these periods reflects the fact that PTSD is known often to be a chronic condition. In a survey of young urban adults 57% of PTSD cases had a duration of more than one year [12], whereas in a major U.S. national survey PTSD failed to remit in more than one third of persons even after many years [7]. In a substantial number of cases PTSD does not even begin until after six months have passed since the index traumatic event [13], and in a U.K. military sample the median lag among those with a delayed onset was 31 months after the event [14]. These considerations are particularly relevant to understanding PTSD and Complex PTSD arising from a career in a police service. Complex PTSD often arises from an accumulation of different stressors occurring over a protracted period of time.

The Policing: The Job and the Life survey enquired about traumatic events occurring during the entire period of employment as a police officer [5]. Unpublished data from the survey confirm the importance of asking about events that occurred more than six months before that are associated with current PTSD and Complex PTSD. In the context of a police career 70% of respondents’ single most upsetting traumatic events had occurred more than six months previously. They were most likely to have occurred between one and five years previously, but might have occurred more than 20 years earlier. Reflecting the complex and prolonged nature of trauma exposure in police officers, some respondents alluded to continual, ongoing trauma (weekly or even daily), or to trauma originating from a variety of different time periods. These observations underscore the difficulty that may be faced in assigning traumatic events to a specific timeframe.

Stevelink et al. (2020) only assessed PTSD in relation to traumatic events occurring in the previous six months, a decision which meant that only 13.5% of their sample were eligible for a PTSD diagnosis. Their figure for the prevalence of PTSD appears to have utilised the number of cases in this eligible sample (1,474) as the numerator and the total sample size (40,299) as the denominator. Because they did not administer the PTSD diagnostic questionnaire to the remaining 86.5% of the sample their prevalence rate is not comparable with other similar surveys. It is reasonable to assume that many cases involving PTSD to an earlier event were missed.

The data from the Policing: The Job & the Life survey were not exactly comparable to those of Stevelink et al. but we have now tried to replicate their analysis as closely as possible by calculating a prevalence rate for PTSD and Complex PTSD combined, based solely on cases where the most troubling experience occurred in the previous six months. This subset amounted to 22.4% of the total sample, rather more than in Stevelink et al.’s survey. The resulting 6-month rate was 27.7% (almost identical to Stevelink et al.’s 27.0%). If we then take this as the basis for calculating the prevalence of PTSD and Complex PTSD in the entire sample, as they did, and adjust for the different proportion of 6-month onsets in the two studies, our rate is now 3.7% (their rate was 3.9%). This demonstrates, in our view, that this aspect of their methods can potentially account for the entire discrepancy between the two studies.

Prevalence rates are based on the total number of cases present in a population at a particular point or period, and include both recent and existing cases. Thus, Stevelink et al.’s (2020) figure of 3.9% specifically reflects the prevalence of recent-onset PTSD within the entire U.K. police force. They also found, replicating the Policing: The Job & the Life survey, that 27% of those with recent traumatic events had a probable PTSD diagnosis. We have demonstrated that their data in no way contradict the recent estimate that the total PTSD and Complex PTSD prevalence in the U.K. police force amounts to 20.6% [3]. The conclusion is that it is important to distinguish carefully the nature of reported prevalence rates so as not to underestimate the potential impact of trauma exposure on the mental health of police officers.
